# Semi-Self-Supervised Learning for Semantic Segmentation in Images with Dense Patterns

**DOI:** 10.34133/plantphenomics.0025

**Published:** 2023-02-24

**Authors:** Keyhan Najafian, Alireza Ghanbari, Mahdi Sabet Kish, Mark Eramian, Gholam Hassan Shirdel, Ian Stavness, Lingling Jin, Farhad Maleki

**Affiliations:** ^1^ Department of Computer Science, University of Saskatchewan, Saskatoon, Saskatchewan, Canada.; ^2^ Mathematics Department, Faculty of Sciences, University of Qom, Qom, Iran.; ^3^ Department of Mathematics, Faculty of Mathematical Science, Shahid Beheshti University, Tehran, Iran.; ^4^ Department of Computer Science, University of Calgary, Calgary, Alberta, Canada.

## Abstract

Deep learning has shown potential in domains with large-scale annotated datasets. However, manual annotation is expensive, time-consuming, and tedious. Pixel-level annotations are particularly costly for semantic segmentation in images with dense irregular patterns of object instances, such as in plant images. In this work, we propose a method for developing high-performing deep learning models for semantic segmentation of such images utilizing little manual annotation. As a use case, we focus on wheat head segmentation. We synthesize a computationally annotated dataset—using a few annotated images, a short unannotated video clip of a wheat field, and several video clips with no wheat—to train a customized U-Net model. Considering the distribution shift between the synthesized and real images, we apply three domain adaptation steps to gradually bridge the domain gap. Only using two annotated images, we achieved a Dice score of 0.89 on the internal test set. When further evaluated on a diverse external dataset collected from 18 different domains across five countries, this model achieved a Dice score of 0.73. To expose the model to images from different growth stages and environmental conditions, we incorporated two annotated images from each of the 18 domains to further fine-tune the model. This increased the Dice score to 0.91. The result highlights the utility of the proposed approach in the absence of large-annotated datasets. Although our use case is wheat head segmentation, the proposed approach can be extended to other segmentation tasks with similar characteristics of irregularly repeating patterns of object instances.

## Introduction

Deep learning models have shown promising results in various computer vision tasks, including object recognition [1], object detection [2], instance segmentation [3], and semantic segmentation [4,5]. Deep learning has the potential to be applied to crop images to inform plant breeding and precision agriculture in order to improve the quality and quantity of crop production [6–12]. Image-based phenotyping of several important plant traits, such as organ size, organ health, and response to biotic and abiotic stress, requires fine-grain semantic segmentation of plant organs [13]. Semantic segmentation in crop images remains a substantial challenge since creating pixel-level annotations for plant images is costly because of the dense, partly occluding, and repeating pattern of plants and plant organs in field images.

Plant and leaf segmentation using deep convolutional neural networks have gained considerable attention in the plant phenotyping research community recently. Ullah et al. [14] developed a segmentation model based on a lightweight dilated U-Net model [15] to segment the crop, weeds, and background in the canola field images. Das et al. [16] developed a model to segment weed, canola, and canola flea beetle damage. To address the high class imbalance, which poses a substantial challenge for segmentation models, they developed a customized deep learning architecture and used a weighted binary cross entropy loss. Hussein et al. [17] developed a deep learning-based segmentation pipeline for leaf segmentation in herbarium specimen images using DeepLabv3+ architecture [18]. Utilizing the connected components algorithm, they split the contour masks into individual leaves. Alkhudaydi et al. [19] utilized a fully convolutional network [20] for wheat spike region segmentation using a small dataset of 90 side-view images. Finally, the availability of large-scale and diverse datasets such as the Global Wheat Head Detection (GWHD) dataset [21,22] has enabled new opportunities for researchers to develop novel supervised deep learning-based methods for wheat phenotyping from field images [11,23–26].

A small number of papers have investigated wheat head segmentation specifically. A few prior works have converted the wheat head segmentation problem to a superpixel classification problem [27–29]. The main idea behind these works is to group pixels into homogeneous superpixels using the simple linear iterative clustering [30]. These superpixels are then classified to determine regions belonging to wheat heads. The superpixel generation is often sensitive to background clutter and illumination condition. Consequently, some superpixels partially cover spike and nonspike regions. This results in coarse segmentation masks. For example, Ubbens et al. [31] proposed AutoCount, an unsupervised approach for the segmentation and counting of plant organs using a linear iterative clustering superpixel segmentation. This segmentation was then used to train a fully convolutional network for organ segmentation. While this approach does not require any manual segmentation, the superpixel segmentation step is a bottleneck of the model performance.

Rawat et al. [32] studied the utility of active learning approaches for wheat head segmentation, and their results represent the current state-of-the-art performance. They investigated pool-based active learning and four methods based on least confidence, margin, entropy, and deep Bayesian for plant organ segmentation in apple, rice, and wheat. For wheat head segmentation, they manually segmented images from the UTokyo_Wheat_2020 dataset, which is a subdomain of the 2020 GWHD. Their model achieved an intersection over union (IoU) of about 0.70 when evaluated on a test set from the same UTokyo domain of the GWHD.

In order to reduce the cost of data annotation, self-supervised learning and semi-supervised learning approaches have been developed for computer vision tasks [33] (see Section S1 in the Supplementary Materials). Recently, Pauletto et al. [34] proposed a neural architecture search strategy utilizing co-teaching, Mean Teacher, and jigsaw puzzle methods for semantic image segmentation. They utilized both annotated and unannotated images. When compared to DeepLabv3+ [18], the author observed a marginal gain using both self-supervised and semi-supervised approaches for one of the datasets under study (Pascal VOC 12). The proposed approach by Pauletto et al. cannot be applied in scenarios where only a handful of annotated images are available. It also does not use the characteristic of the images with dense and self-similar patterns, which are common in image-based plant phenotyping. Fourati et al. [23] developed a wheat head detection method based on Faster R-CNN [35], and EfficientDet [36] models using the GWHD dataset as the training dataset. They also utilized pseudo labeling, which is considered a semi-supervised approach, alongside test time augmentation (TTA), multiscale ensemble, and bootstrap aggregation to further increase the model performance, achieving a mean average precision of 0.74. Najafian et al. [24] proposed a deep learning-based approach for wheat head detection by developing a simulated dataset using a cut-and-paste approach [37]. They trained a YOLO architecture [38] for wheat head detection using the simulated dataset and then fine-tuned the model with the GWHD dataset. The model achieved a mean average precision of 0.82. While their approach facilitated the development of a deep learning-based wheat head detection model, their model achieved low performance when trained only using their simulated dataset.

**Fig. 1.**
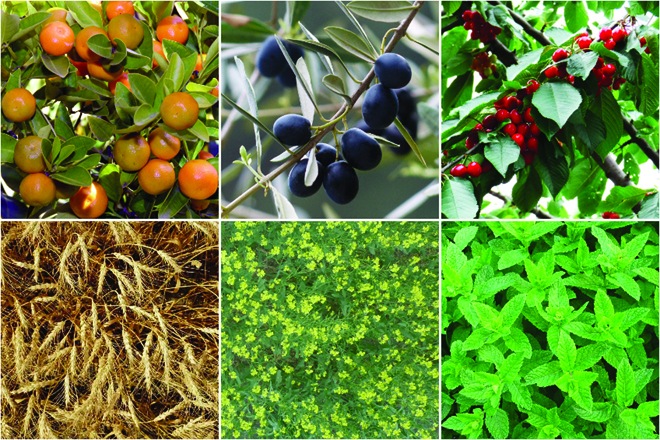
In agricultural images, we are often interested in densely packed, overlapping, and highly self-similar instances. In these applications, images often contain irregular patterns such as plant spikes, fruits, flowers, or leaves.

Most segmentation tasks in plant phenotyping share characteristics that make them different from segmentation tasks for general images such as those developed using Pascal VOC [39] and MS COCO [40] datasets. Unlike general object segmentation, plant phenotyping tasks involve segmenting densely packed and overlapping instances that are highly self-similar. In such applications, images often contain many repeated irregular patterns, such as plant spikes, fruits, flowers, or leaves. Figure 1 illustrates a few examples of such images. These distinctive features pose challenges and opportunities that should be considered when developing segmentation models in agricultural domains.

Considering these characteristics of crop images, in this paper, we propose an image synthesis and model training pipeline to develop semantic segmentation models with very small number of annotated images. Our method utilizes a short video clip of a wheat field, a few annotated images, and several short video clips of background scenes—i.e., fields with no wheat—to synthesize a large-scale computationally annotated dataset. Then, using this synthesized dataset, a customized U-Net model for wheat head segmentation is trained in a supervised manner. To bridge the domain gap between synthesized and real images, three domain adaptation steps are applied. We evaluate the proposed method using the GWHD dataset. The proposed approach alleviates the need for creating a costly large-scale annotated dataset and facilitates the accelerated development of deep learning models for plant phenotyping.

## Materials and Methods

In this section, we present our methodology for developing wheat head segmentation models utilizing a small amount of annotated images. We first synthesize a large-scale computationally annotated dataset using a few annotated images. The synthesized images are then used to train a customized U-Net model for wheat head segmentation. Because of the difference between the synthesized and real images, also known as distribution shift, we expect the performance of the model trained on synthetic images to degrade when applied to real images. To address this domain gap, we apply three domain adaptation steps. We evaluate the proposed method using a diverse collection of wheat field images. In the following, we provide a detailed description of (a) data used for experiments, (b) process for data synthesis, (c) model building, and (d) domain adaptation steps.

### Data

**Fig. 2.**
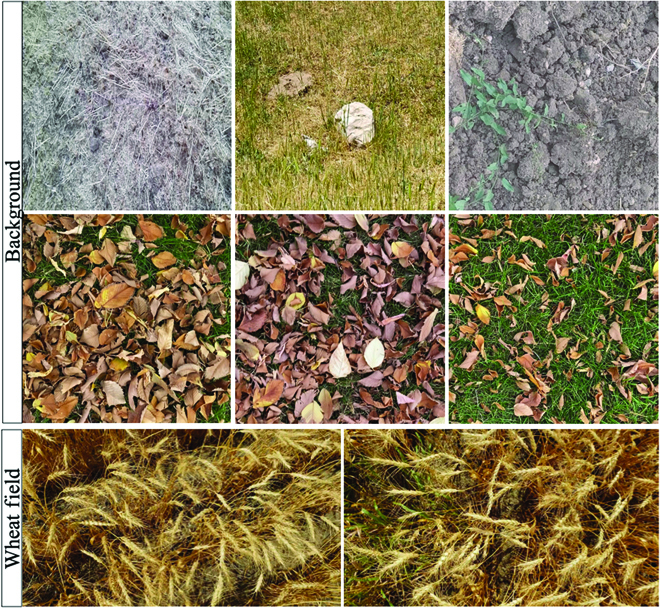
The top two rows show extracted image frames from the background video clips—i.e., video clips of fields with no wheat plants. The third row shows examples of image frames extracted from the video clip of a wheat field.

**Fig. 3.**
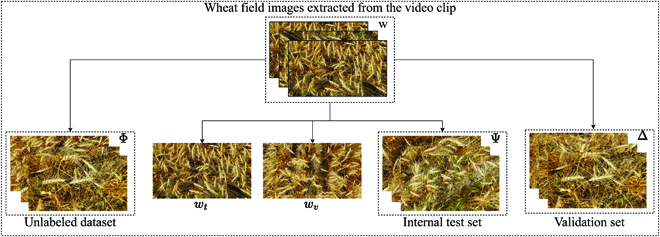
The image frames from the wheat field video clip are partitioned to five subsets: (a) An image *w_t_* is selected to synthesize a training set of images. (b) An image *w_v_* is selected to synthesize a validation set of images. (c) A set Ψ of 100 images that are randomly selected and manually annotated to be used as an internal test set. Images of Ψ are selected such that they are not within one second from *w_t_* and *w_v_*. (d) A set Δ of 10 images selected and used as a validation set. (e) The rest of the images are used as an unannotated set of images, Φ.

The data used for this study is available at https://www.cs.usask.ca/ftp/pub/whs/. We utilize a short video clip of a wheat field and 11 short video clips of background scenes to synthesize annotated images. These video clips were obtained using Samsung cameras with 12 and 48-megapixel resolution. Figure 2 illustrates snapshots of some video clips. Image frames of wheat scene and background scene from these video clips are extracted and used for the experiments. We refer to the set of all image frames from the video clip of the wheat field as *W*. We partition *W* into five groups (see Fig. 3): (a) An image *w_t_* is selected to synthesize a training set of images. (b) An image *w_v_* is selected to synthesize a validation set of images. (c) A set Ψ of 100 images that are randomly selected and manually annotated to be used as an internal test set. Images in Ψ are selected in such a way that they are not within one second from *w_t_* and *w_v_*. (d) A set Δ of 10 images selected and used as a validation set. (e) The rest of the images are used as unannotated set of images Φ.

**Fig. 4.**
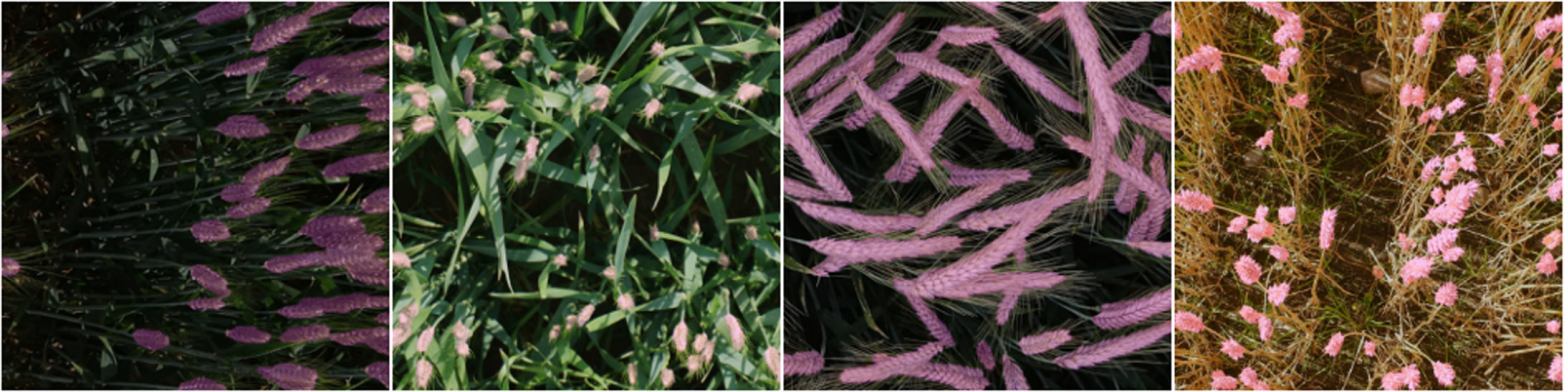
Examples of images in Γ, manually annotated as the external test set. The segmentation masks are overlaid on the images.

In addition to the internal test set, we perform external evaluation using the training set of the GWHD dataset [22], which includes images of wheat fields from five countries and 18 different domains from various plant growth stages. Since there is no segmentation annotation for this dataset, we randomly select a set Γ of 10% of images (365 images) from the training subset of the GWHD dataset and manually segment them for model evaluation. Figure 4 illustrates examples of the images from Γ with their segmentation masks overlaid.

From the remaining images in the training set of GWHD dataset, we randomly select 36 images (two images from each of the 18 domains) and manually segment them. This set of images is partitioned into two subsets Θ*_t_* and Θ*_v_*, so that each subset contains one sample from each domain. We also add *w_t_* to Θ*_t_* and *w_v_* to Θ*_v_*. Images in Θ*_t_* and Θ*_v_* are used for the final step of domain adaptation (see below). In this paper, we use subscripts *t* and *v* to represent training and validation sets, respectively.

For model comparison, we also use the UTokyo subset of the GWHD dataset to build a set Λ of 100 images to compare our model with the wheat head segmentation model developed by Rawat et al. [32]. The UTokyo dataset has three subgroups (UTokyo_1, UTokyo_2, and UTokyo_3). Λ is built by randomly selecting 100 images from these three subsets, providing almost equal representation for each subgroup.

### Image synthesis

**Fig. 5.**
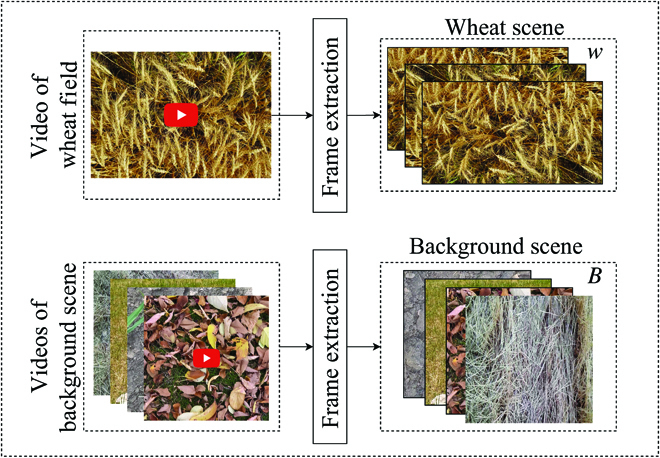
Extracting image frames from videos to build a set of wheat field images (*W*) and a set of background images (*B*).

We synthesize a dataset of wheat field images using video clips of wheat fields and background scenes. The code used for image synthesis is available at https://github.com/KeyhanNajafian/ImageSimulatorPipeline. As shown in Fig. 5, we extract *m* background images *B* = {*b*_1_, *b*_2_, …, *b_m_*} from the video clips of the background fields and *n* wheat field images *W* = {*w*_1_, …, *w_n_*} from the wheat field video clip. These images are used to synthesize a computationally annotated dataset in two steps as follows.

**Fig. 6.**
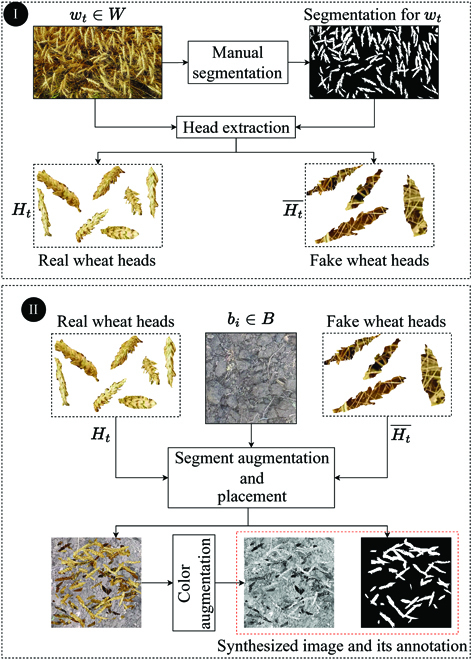
The procedure for synthesizing the computationally annotated datasets *S_t_*. (I) First, a single image *w_t_* is manually segmented. Then, the wheat heads in *w_t_* are extracted to build set *H_t_*. Next, to build a set of fake wheat heads $\bar{H_{t}}$, the shapes of extracted wheat heads are used as cookie cutters to extract regions of *w_t_* that do not contain wheat heads. (II) A background image *b_i_* from the set of all image frames extracted from background videos is selected and used as a canvas. First, a set of fake wheat heads from $\bar{H_{t}}$ is selected and overlaid on *b_i_*. Then, a set of wheat heads from *H_t_* are selected and transformed. The transformed wheat heads are overlaid on *b_i_*, and their position is recorded to computationally generate the annotation for the synthesized image. The resulting image undergoes a sequence of color augmentations.

Step I: Select two different wheat field images, *w_t_* and *w_v_*, to synthesize the training and validation sets, respectively. As depicted in Fig. 6I, these two images are manually segmented. Then, the wheat heads from *w_t_* and *w_v_* are extracted to create two sets *H_t_* and *H_v_*, which are referred to as real wheat heads. We also generate $\bar{H_{t}}$ by using the wheat head shapes in *H_t_* as cookie cutters to extract regions of *w_t_* that do not contain wheat heads. $\bar{H_{v}}$ is generated in a similar way. We refer to $\bar{H_{t}}$ and $\bar{H_{v}}$ as fake wheat heads.

Step II: Synthesize annotated datasets *S_t_* and *S_v_* as illustrated in Fig. 6II. To Synthesize *S_t_*, we select a background image *b_i_* from *B*. This image serves as a canvas where we overlay: (a) a set of fake wheat heads from $\bar{H_{t}}$ and (b) a set of real wheat heads from *H_t_*, selected on the basis of random sampling with replacement. The number of fake and real wheat heads to be randomly placed on *b_i_* is chosen as uniformly distributed random numbers between 10 and 100. We first overlay fake wheat heads and then real wheat heads. Also, prior to placement on *b_i_*, both real and fake wheat heads undergo a sequence of image augmentations including horizontal and vertical flips, rotation, resizing, and elastic transformation [41]. The locations of the real wheat heads are recorded to generate the segmentation mask for the synthesized image. Last, a sequence of color augmentations [42] are applied to the resulting image. This image is then added to *S_t_*. This process is repeated 10,000 times to generate 10,000 synthesized images in *S_t_* for model training. A synthesized dataset *S_v_* of size 1000 is generated analogously to be used for model evaluation.

### Model

In this study, we use a customized U-Net [15] model architecture with the EfficientNet B4 [43] encoder pretrained on the ImageNet dataset [44,45]. The information about network architecture and the machine used for model development is described in Section S3 of the Supplementary Materials. For all experiments in this paper, we employ a loss function that is the summation of Binary Cross-entropy and Dice loss functions, as follows:

$$\begin{array}{c} \text{Loss}=\frac{1}{\left\|\Omega \right\|}\sum_{\Omega_{i}\in \Omega }(1-\frac{(2\sum_{x\in \Omega_{i}}p(x)g(x))+\epsilon }{(\sum_{x\in \Omega_{i}}p(x)+\sum_{x\in \Omega_{i}}g(x))+\epsilon }-\\ \frac{1}{\left\|\Omega_{i}\right\|}\times \sum_{x\in \Omega_{i}}(g(x)\log(p(x))+(1-g(x))\log(1-p(x)))), \end{array}$$ (1)

where *p*(*x*) is the probability of the pixel *x* being part of a wheat head on an image. *p*(*x*) is calculated by applying the sigmoid function to the output of the final convolution layer of the model. The function *g*(*x*) ∈ {0, 1} represents the ground truth class for pixel *x*, which is 1 if *x* is part of a wheat head and 0 otherwise. Ω is a batch of images in the training set, and Ω*_i_* is an image in Ω undergoing a sequence of image transformations with computationally inferable labeling functions. ∥Ω∥ represents the number of images in a batch of images Ω, and ∥Ω*_i_*∥ represents the number of pixels in an image Ω*_i_*. Also, *ϵ* is a smoothing constant to prevent division by zero. We used *ϵ* = 10^−5^ for all experiments.

The computationally inferable labeling function is formulated as follows. Assume that for a data point *x* in a domain *X*, there is a labeling function *f* that assigns a label *y* to *x*, that is, *f*(*x*) = *y*. Also, assume that $T:X\to \tilde{X}$ is a transformation function that maps each data point *x* from domain *X* to a data point $\tilde{x}$ from domain $\tilde{X}$. The labeling function *f* for a transformation *T* is computationally inferable if for any *x* ∈ *X*, there is a deterministic algorithm that outputs *f*(*T*(*x*)), which is the label for $\tilde{x}=T\left(x\right)$. Note that we differentiate a sequence of image augmentations from a sequence of image transformations with computationally inferable labeling functions, as the former does not always lead to an image where labels can be inferred. For example, through the image augmentations, an image might get so distorted that, even for an expert, manual labeling is not possible. We use the Albumentations package version 1.1.0 [42] for all image augmentations in this study.

We also use a TTA approach to improve the predictions made by the model. During this process, for each image, we generate two augmented versions using Gaussian noise and Sepia image augmentations [42]. Then, the predictions made by the model for the original image and its two augmented versions are aggregated using a pixel-level majority vote to compute the final prediction.

In this study, we utilize the commonly used Dice score and IoU as our performance measures. Given an observed segmentation mask *O* and the expected segmentation mask *E*, the Dice score is defined as:

$$\text{Dice}\left(O,E\right)=\frac{2∥O\cap E∥}{∥O∥+∥E∥}$$ (2)

where ∥∥ and ∩ represent set cardinality and intersection operators, respectively. IoU is defined as follows:

$$\mathrm{IoU}\left(O,E\right)=\frac{∥O\cap E∥}{∥O\cup E∥}$$ (3)

where ∪ represents the union operator.

In all experiments, we use the smallest loss value on the validation sets as the criterion for model selection. We also use the SGD optimizer [46] with a learning rate of 0.01.

### Domain adaptation

Often, the performance of a model degrades when it is applied to a dataset with a distribution different from the distribution of data used for its development. This change in data distribution is referred to as a distribution shift or domain shift. Since there is a domain shift between the images in the synthesized datasets *S_t_* and *S_v_* and real images, domain adaptation is required to improve the model performance. In this study, we apply three domain adaptation steps to progressively fine-tune the model, resulting in a new model after each step, denoted as *D*, *P*, and *G*. The details of domain adaptation steps are described as follows.

**Fig. 7.**
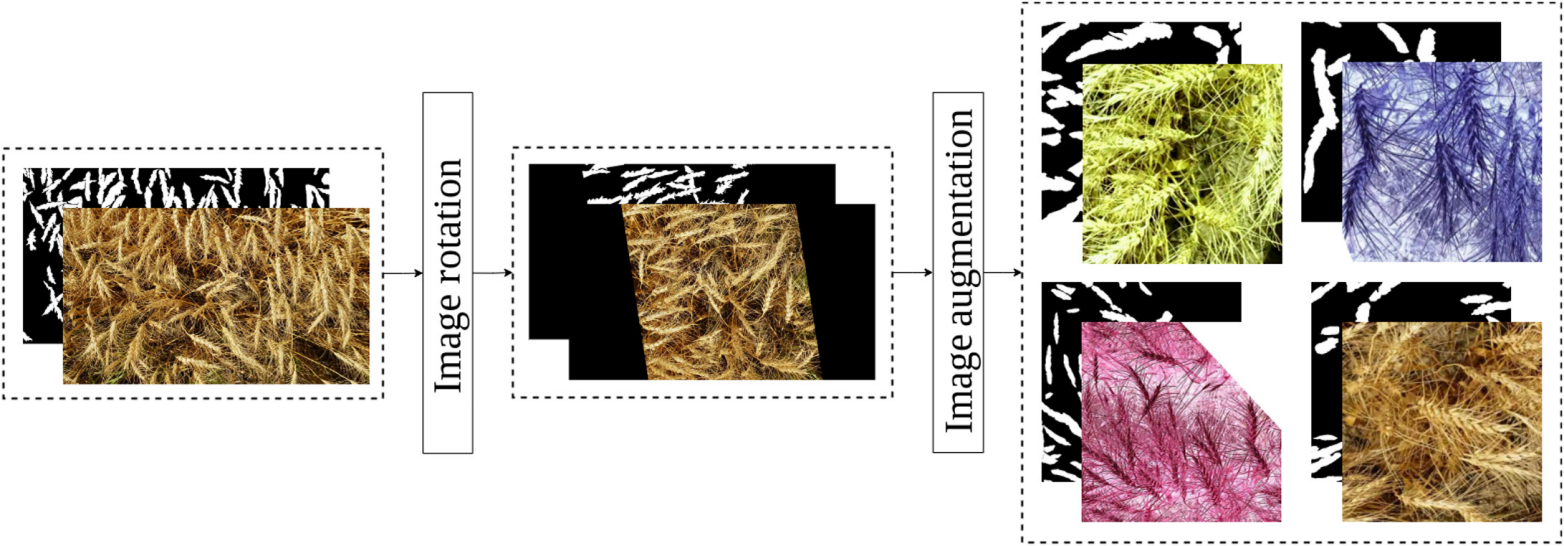
The procedure for generating datasets *D_t_* and *D_v_* for the first domain adaptation step.

**Fig. 8.**
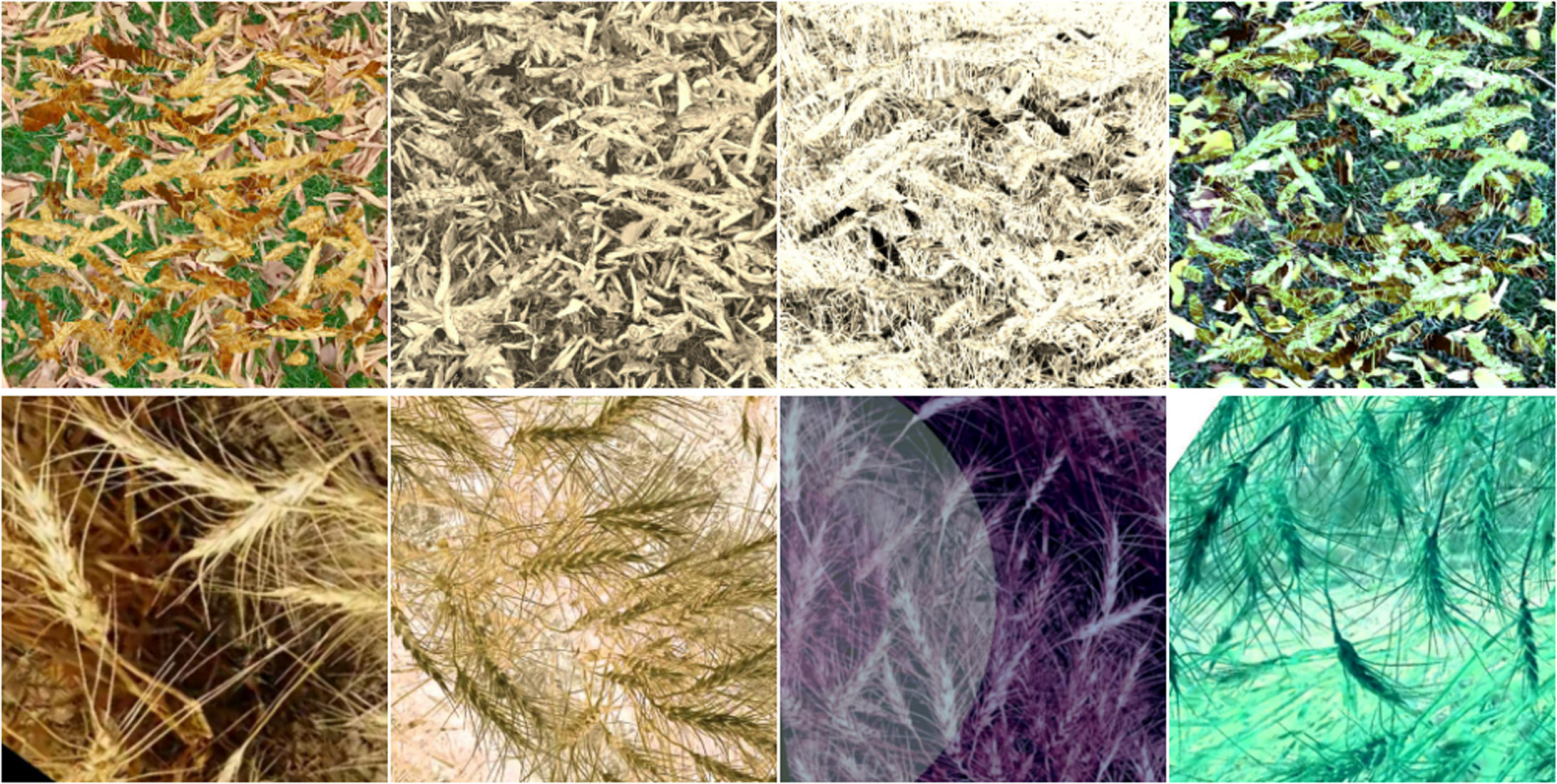
The top row illustrates examples of synthesized images (dataset *S_t_*), and the bottom row shows examples of strongly augmented images (dataset *D_t_*) generated in the first domain adaptation step.

Step I: **Model *D***. As the first step, we develop datasets *D_t_* and *D_v_* and use these datasets to fine-tune the model *S*. The images in set *D_t_* and *D_v_* are real images with some transformations applied. The choice of transformation has been made in a way that the manual annotation of the resulting images is possible. Unlike images in *S_t_* and *S_v_*, these images show the whole structure of wheat plants and their positional characteristics. Therefore, images in *D_t_* and *D_v_* are semantically more similar to real images. Figure 7 illustrates the process for generating images in datasets *D_t_* and *D_v_*. These datasets are generated by utilizing all 360 rotations (from 0 to 359 degrees) of *w_t_* and *w_v_*, respectively. These rotated images undergo a comprehensive set of image augmentations, which will be applied in an online manner—i.e., each time we access an image, a different sequence of image augmentations is applied to the image. These augmentations are applied to increase the data variability and avoid overfitting. Figure 8 offers a visual comparison between images in *S_t_* and *D_t_*. Section S2 in the Supplementary Materials provides the list of image augmentations applied to images in *D_t_* and *D_v_*. We refer to the model trained with these datasets as model *D*.

Step II: **Model *P***. In this step, we apply pseudo labeling. Using model *D*, we predict the segmentation mask for the images in Φ. We refer to these images as pseudo-labeled images since their masks might be noisy because of the model error. The resulting pseudo-labeled images in Φ and images in Δ are used as the training and validation sets, respectively, to fine-tune model *D*. We refer to this fine-tuned model as model *P*.

Step III: **Model *G***. In the third domain adaptation step, we utilize Θ*_t_* and Θ*_v_* to further address the domain shift between synthesized and real images. We expand each set by including all 359 rotations of its images. The resulting sets are used to fine-tune model *P*. We refer to the fine-tuned model as model *G*.

**Fig. 9.**
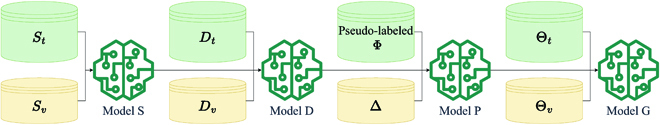
A customized U-Net [15] model architecture with the EfficientNet B4 [43] encoder used for all experiments. For the initial model, we used the backbone pretrained on the ImageNet. The pretrained model from each step is further trained using the designated training and validation sets. Training sets are represented with green cylinders, and the validation sets are depicted with yellow cylinders. *S_t_* and *S_v_* are synthesized datasets. *D_t_* and *D_v_* are used in the first step of domain adaptation. Φ and Δ are image frames from the wheat field video clip, and Θ*_t_* and Θ*_v_* are images from the Global Wheat Head Detection (GWHD) dataset.

Figure 9 summarizes the model development process, in which we use the datasets *S_t_* and *S_v_* to develop model *S*. This model is then fine-tuned using datasets *D_t_* and *D_v_* to develop model *D*. Next, we further train model *D* using the pseudo-labeled datasets Φ (for training) and Δ (for validation) to develop model *P*. Finally, we fine-tune model *P* using datasets Θ*_t_* and Θ*_v_* to develop model *G* as the final model.

As a baseline model using a conventional supervised learning approach, we also developed a model using Θ*_t_* and Θ*_v_* alone. To train the model, we employed the same model architecture with the pretrained backbone on ImageNet. For the sake of comparison, we used the same hyperparameters and image augmentations that we used in developing model *G*. We refer to this model as the Baseline model.

## Results

**Fig. 10.**
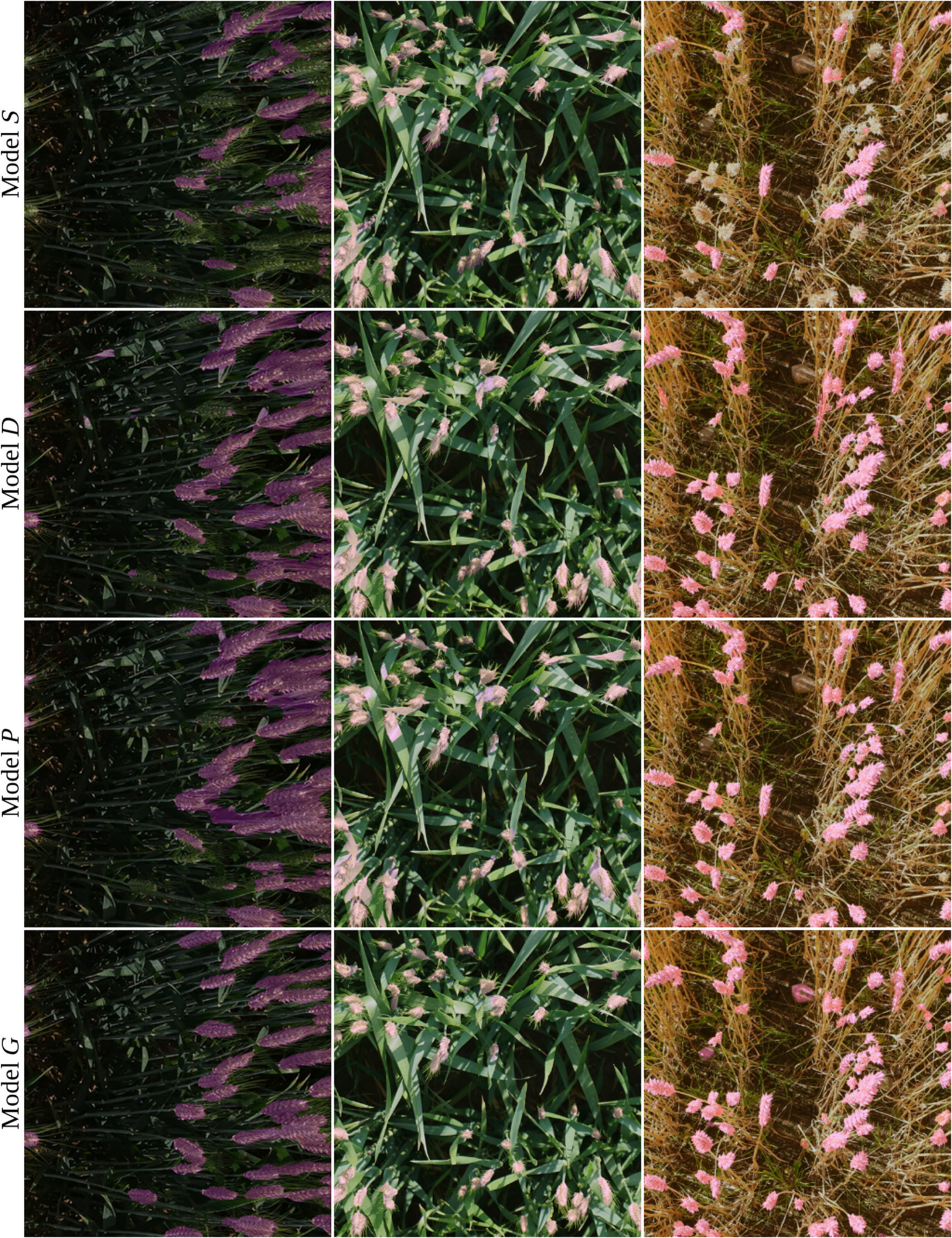
Examples of images from the GWHD dataset predicted by different models. The predicted masks are overlaid on the images. The images in each row shows the prediction made by a model. Model *S* is the model trained on the synthesized dataset. Models *D*, *P*, and *G* are the results for the first, second, and third domain adaptation steps.

Performance measures for all models on the three test sets are reported in Table 1. Figure 10 illustrates the predictions for models *S*, *D*, *P*, and *G* for three randomly chosen images from the GWHD dataset. These images have not been used for model development. TTA (Table 1 TTA Dice column) provided a small improvement over the trained models (Table 1 Dice column) in most, but not in all cases. Performance trends were similar between Dice and IoU measures. Training with the addition of augmented real images (model *D*) provided a consistent improvement over synthetic images alone (model *S*) in all evaluations. Training with additional pseudo-labeled images from the video clip (model *P*) compared to model *D* with TTA, resulted in the same performance on the internal test set, and slightly degraded performance on the external test sets. However, in all comparisons, model *P* without TTA resulted in a slight improvement compared to model *D*. This suggests that model *P* is less robust against the image variations introduced in TTA. The model developed using two annotated images from the wheat field clip along with the synthesis, augmentation, and TTA (model *D*) achieved a TTA Dice score of 0.88 for the internal test set Ψ images from the same wheat field.

**Table 1.** The IoU and Dice score for the Baseline model, the model *S*, which is trained on the synthesized datasets, as well as models *D*, *P*, and *G*, which are the models resulting from the first, second, and third domain adaptation steps. We provide performance measures on test sets, Ψ, Γ, and Λ. These performance measures are calculated for these models with and without test time augmentation (TTA). ImageNet refers to the model with its backbone pretrained on the ImageNet dataset.

Experiment	Initial model	Test set	Dice	IoU	TTA Dice	TTA IoU
Baseline	ImageNet	Ψ(Internal)	0.6358	0.4706	0.4725	0.3150
Model *S*	ImageNet		0.7090	0.5658	0.7804	0.6463
Model *D*	Model *S*		0.8860	0.7974	0.8822	0.7910
Model *P*	Model *D*		0.8984	0.8167	0.8864	0.7975
Model *G*	Model *P*		0.8902	0.8038	0.8871	0.7986
Baseline	ImageNet	Γ(GWHD 18 domains)	0.5080	0.3698	0.4616	0.3235
Model *S*	ImageNet		0.3678	0.2742	04845	0.3745
Model *D*	Model *S*		0.6528	0.5272	0.7416	0.6225
Model *P*	Model *D*		0.6748	0.5606	0.7289	0.6165
Model *G*	Model *P*		0.9144	0.8583	0.9084	0.8489
Baseline	ImageNet	Λ(GWHD UTokyo)	0.4067	0.2749	0.3289	0.2031
Model *S*	ImageNet		0.7553	0.6361	0.8046	0.6998
Model *D*	Model *S*		0.8396	0.7545	0.8037	0.7054
Model *P*	Model *D*		0.8421	0.7657	0.7961	0.6957
Model *G*	Model *P*		0.9270	0.8878	0.9189	0.8742

When testing model *P* with the external test set Γ, the TTA Dice score dropped to 0.73. This highlights the need to address the substantial domain shift caused by variation in factors such as different wheat varieties, wheat growth stages, illumination conditions, and imaging hardware and techniques. When fine-tuned on 38 annotated and augmented images from the GHWD subdomains under consideration (model *G*), the model performance improved substantially, to a TTA Dice score of 0.91. Model *G* performed equally well (TTA Dice 0.92) when evaluated on Λ, extracted from the UTokyo subdomains of the validation set of the GWHD dataset. This is notable because the UTokyo subdomains were not used for model development; therefore, Λ is considered an external test for model *G*.

Performance measures for 18 individual domains of the Γ test set are reported in Table 2. These results were obtained after applying the TTA on each domain separately. TTA Dice scores for the fine-tuned model (model *G*) were at or above 0.95 for seven of the domains and at or above 0.90 for another six of the domains. The lowest performance was found for the Arvalis_4 and Arvalis_5 domains (TTA Dice of 0.82 and 0.75, respectively). These results highlight the variation in model performance across different domains. The performance variation may be attributed to increased difficulty in specific domains, for example, the worst performing Arvalis_5 domain comprises images with greater amount of blur and occlusion than other domains.

**Table 2.** The performance of the models after applying the TTA on each of the 18 domains of the GWHD dataset. Model *S* is the model trained using the synthesized dataset. Models *D* and *P* are the models resulting from the first and second domain adaptation steps. Model *G* is the resulting model after the third domain adaptation step, i.e., the model pretrained on model *P*, and fine-tuned on the 18 images selected from the GWHD dataset (one per domain).

Domain	Model	Pretrained	Dice score	Domain	Model	Pretrained	Dice score
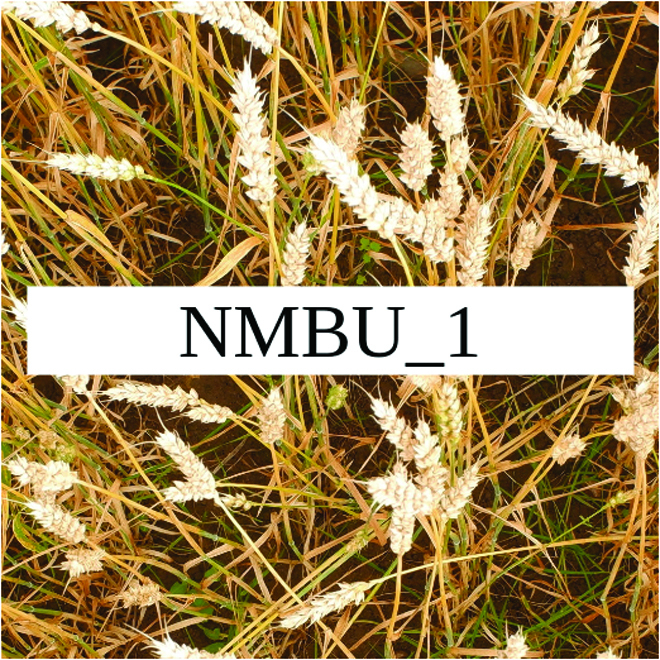	Model *S*	ImageNet	0.7312	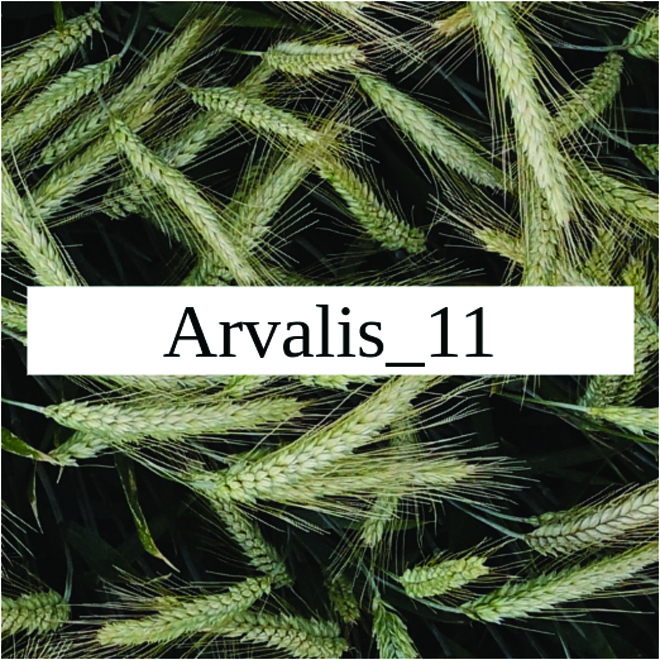	Model *S*	ImageNet	0.7118
	Model *D*	Model *S*	0.9014		Model *D*	Model *S*	0.8739
	Model *P*	Model *D*	0.9114		Model *P*	Model *D*	0.8685
	Model *G*	Model *P*	0.9455		Model *G*	Model *P*	0.9591
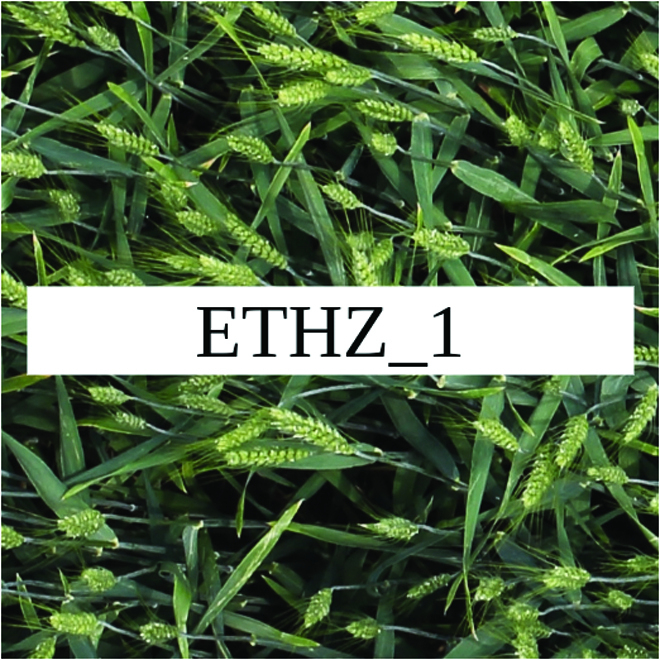	Model *S*	ImageNet	0.8480	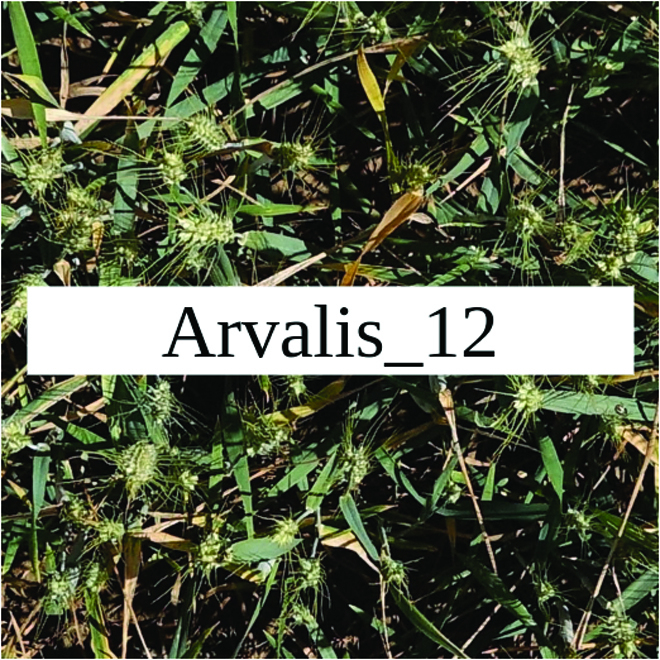	Model *S*	ImageNet	0.2902
	Model *D*	Model *S*	0.9213		Model *D*	Model *S*	0.4046
	Model *P*	Model *D*	0.9297		Model *P*	Model *D*	0.3098
	Model *G*	Model *P*	0.9507		Model *G*	Model *P*	0.7498
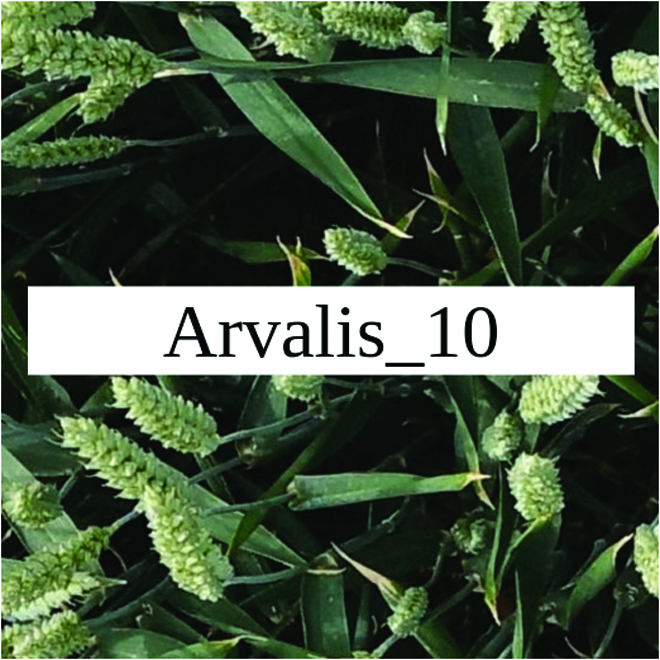	Model *S*	ImageNet	0.3099	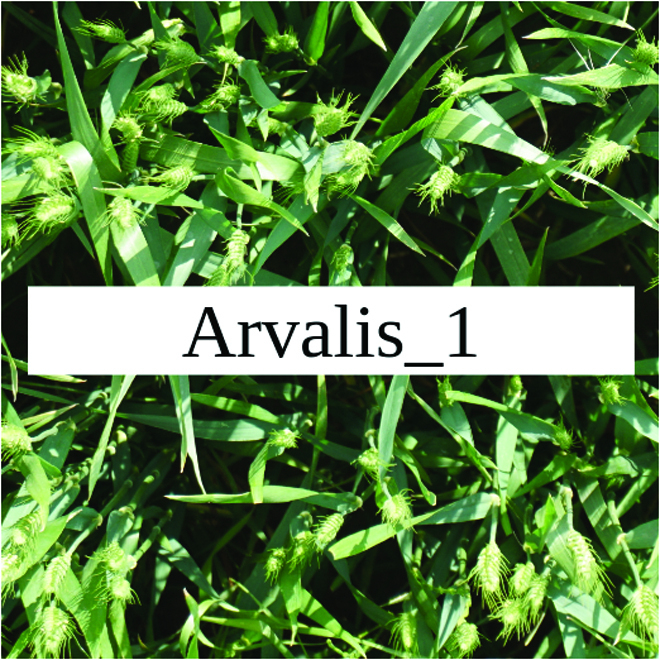	Model *S*	ImageNet	0.6012
	Model *D*	Model *S*	0.7896		Model *D*	Model *S*	0.8268
	Model *P*	Model *D*	0.7971		Model *P*	Model *D*	0.8045
	Model *G*	Model *P*	0.9599		Model *G*	Model *P*	0.9518
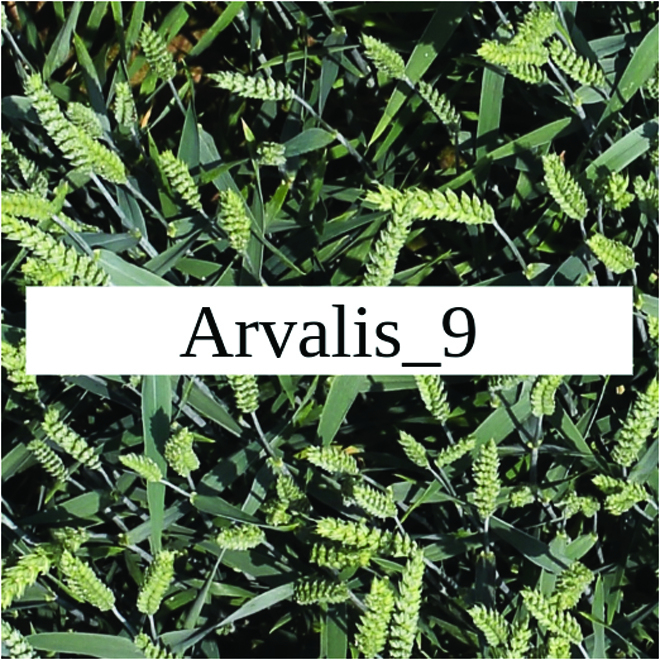	Model *S*	ImageNet	0.2400	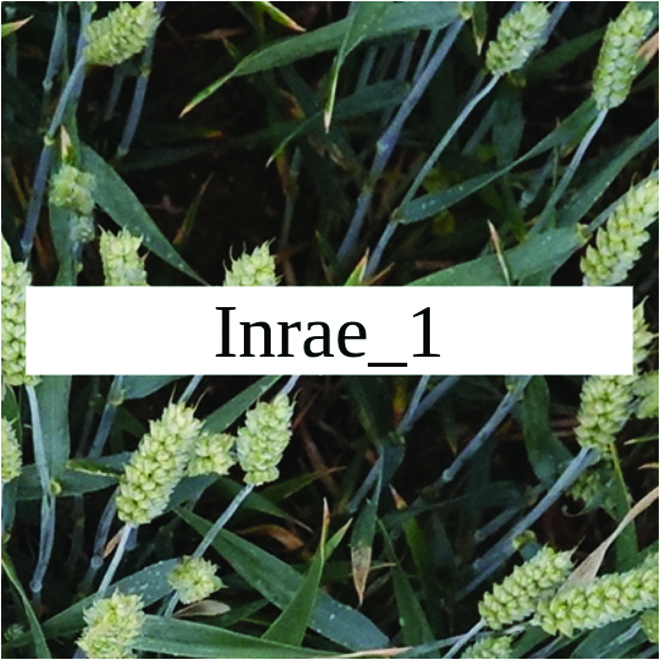	Model *S*	ImageNet	0.5839
	Model *D*	Model *S*	0.7012		Model *D*	Model *S*	0.8120
	Model *P*	Model *D*	0.6428		Model *P*	Model *D*	0.7725
	Model *G*	Model *P*	0.8785		Model *G*	Model *P*	0.9308
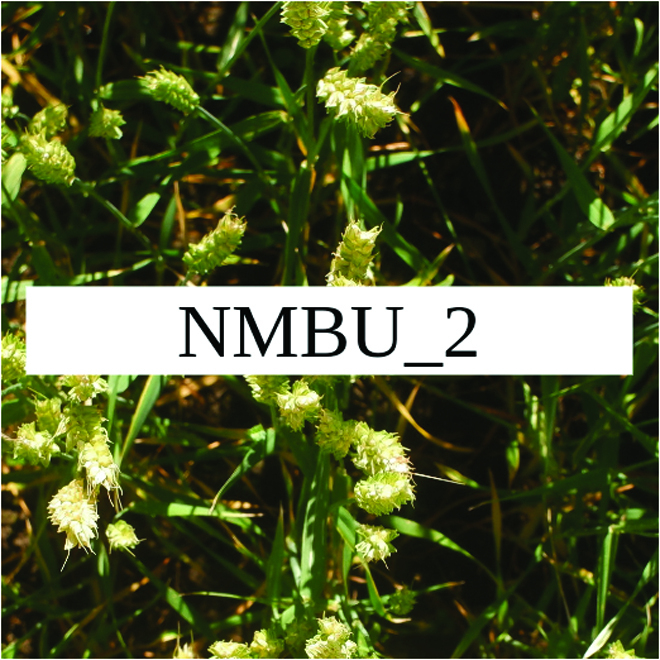	Model *S*	ImageNet	0.7949	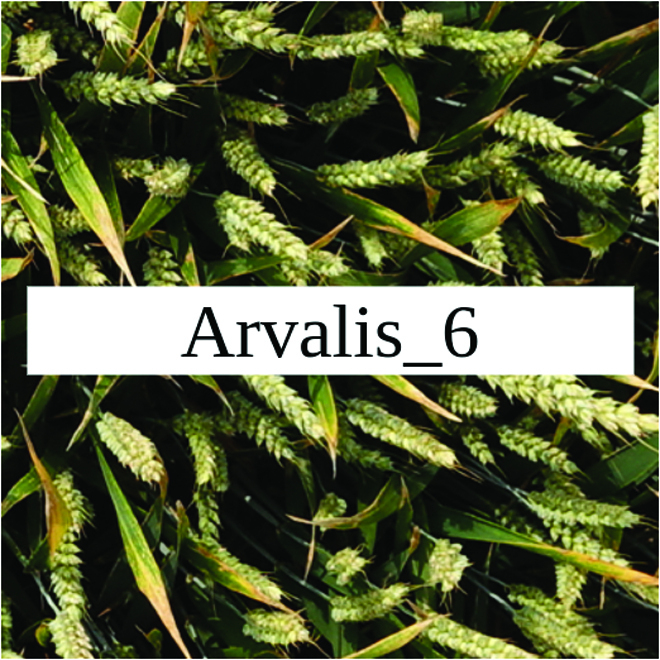	Model *S*	ImageNet	0.1569
	Model *D*	Model *S*	0.8854		Model *D*	Model *S*	0.4745
	Model *P*	Model *D*	0.8886		Model *P*	Model *D*	0.4292
	Model *G*	Model *P*	0.9662		Model *G*	Model *P*	0.8170
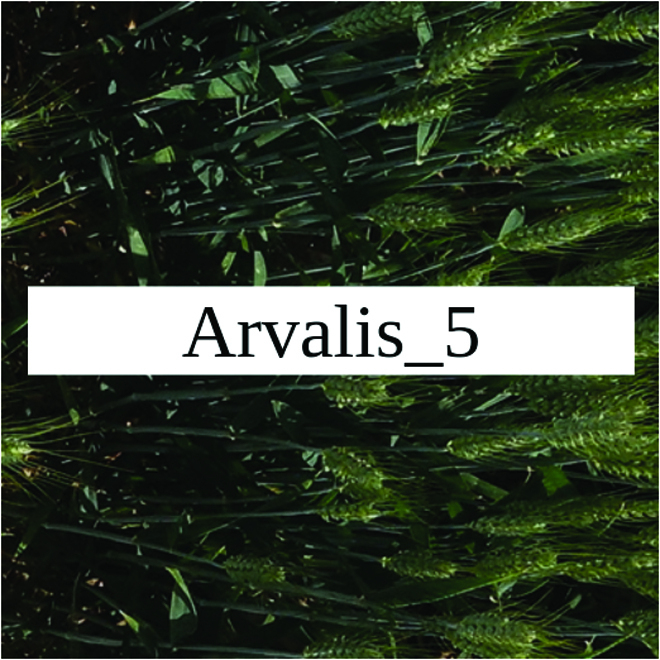	Model *S*	ImageNet	0.3896	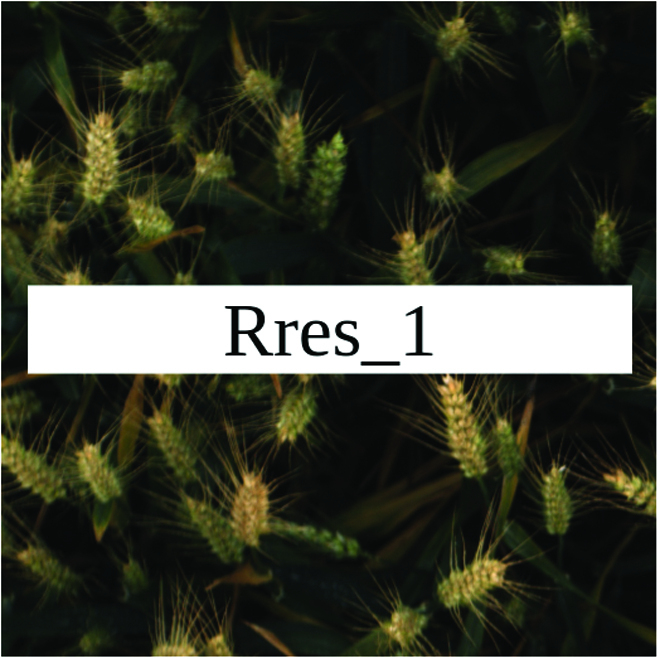	Model *S*	ImageNet	0.5824
	Model *D*	Model *S*	0.6637		Model *D*	Model *S*	0.8088
	Model *P*	Model *D*	0.6143		Model *P*	Model *D*	0.8262
	Model *G*	Model *P*	0.8960		Model *G*	Model *P*	0.9197
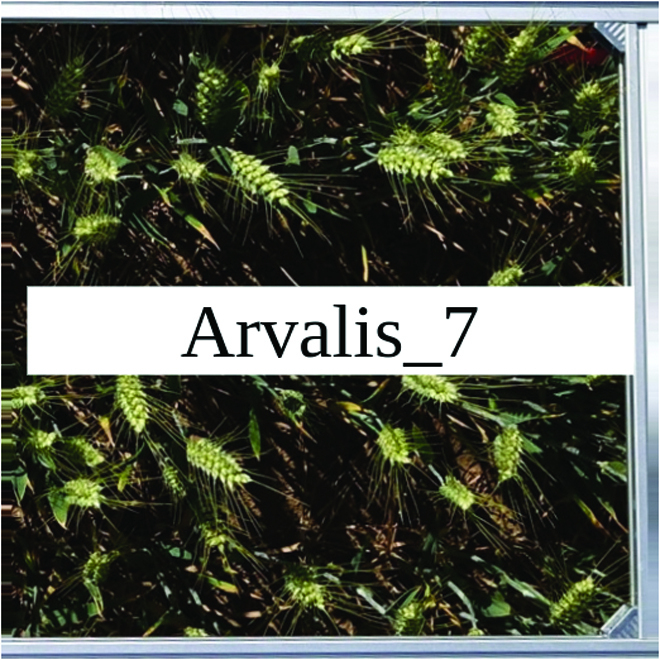	Model *S*	ImageNet	0.5097	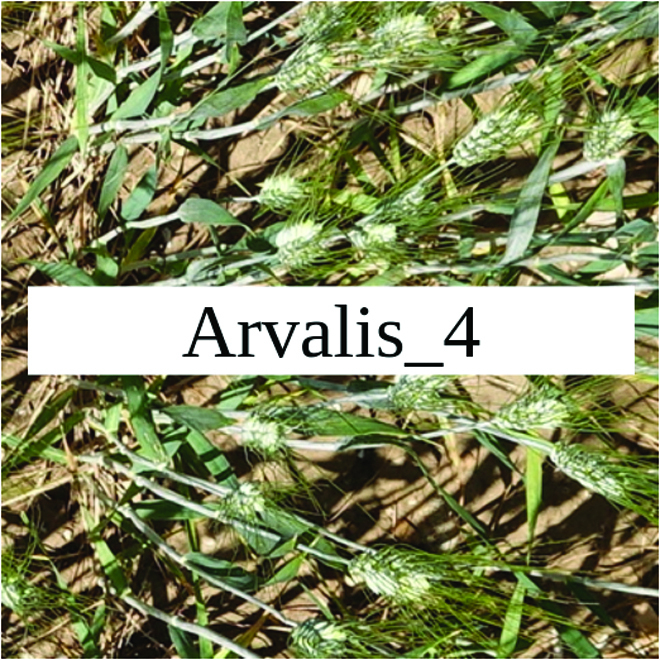	Model *S*	ImageNet	0.8843
	Model *D*	Model *S*	0.7864		Model *D*	Model *S*	0.8664
	Model *P*	Model *D*	0.8078		Model *P*	Model *D*	0.8365
	Model *G*	Model *P*	0.9165		Model *G*	Model *P*	0.9309
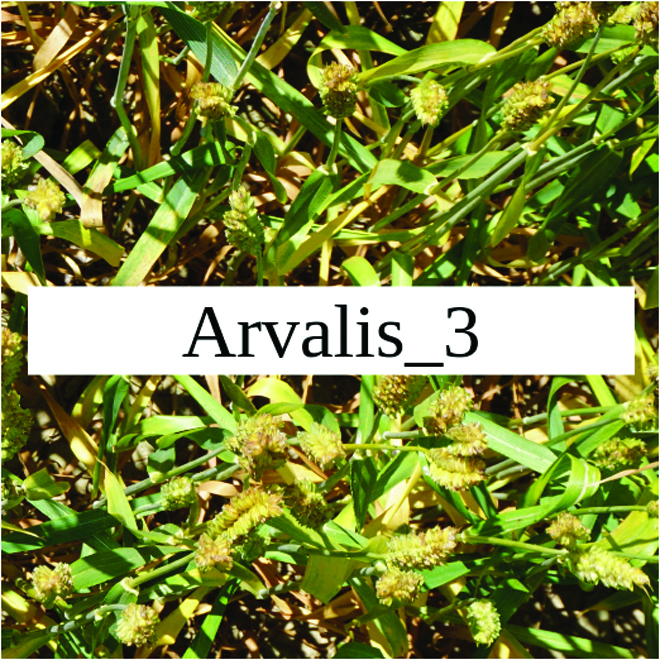	Model *S*	ImageNet	0.8592	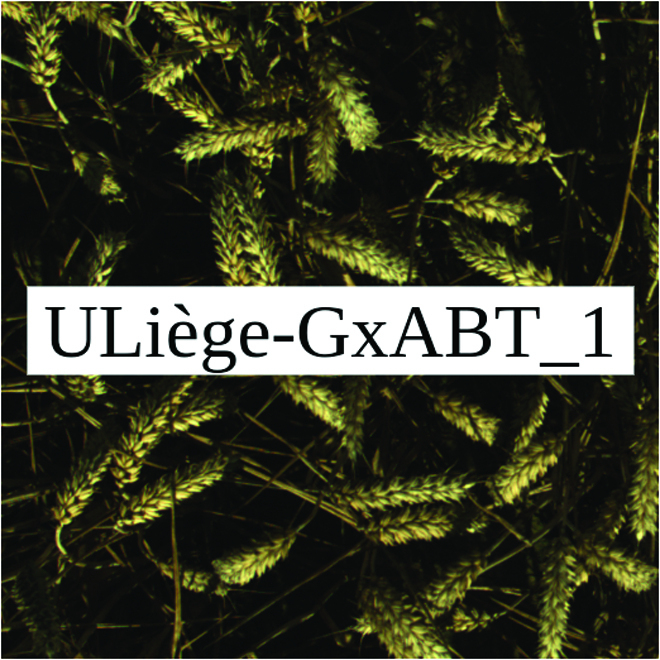	Model *S*	ImageNet	0.5015
	Model *D*	Model *S*	0.8733		Model *D*	Model *S*	0.7682
	Model *P*	Model *D*	0.9148		Model *P*	Model *D*	0.7645
	Model *G*	Model *P*	0.9558		Model *G*	Model *P*	0.9140
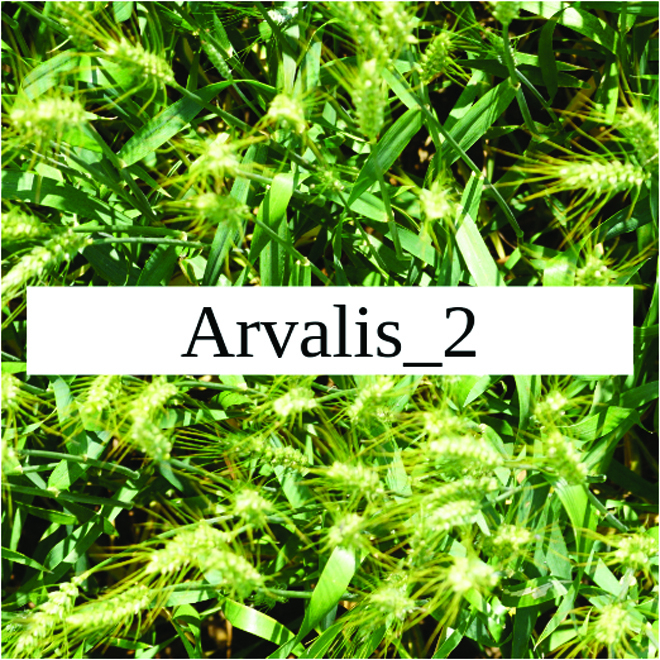	Model *S*	ImageNet	0.5391	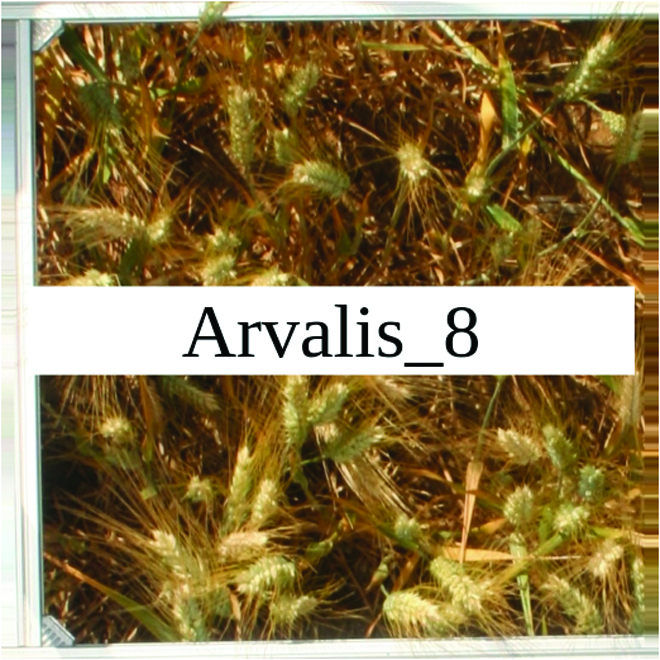	Model *S*	ImageNet	0.6296
	Model *D*	Model *S*	0.7637		Model *D*	Model *S*	0.7935
	Model *P*	Model *D*	0.7772		Model *P*	Model *D*	0.7155
	Model *G*	Model *P*	0.8574		Model *G*	Model *P*	0.9233

## Discussion

In this study, we proposed a semi-self-supervised learning approach to tackle the wheat head semantic segmentation problem. Our approach, based on synthesizing computationally annotated datasets followed by domain adaptation steps, makes it possible to develop a high-performing semantic segmentation model for wheat head segmentation using only two manually annotated images per domain. The proposed method is a self-supervised approach because it relies on developing computationally annotated data for model development. It is also a semi-supervised learning approach because it utilizes a few manually annotated images as well as pseudo labeling for model training. This approach enables the adoption of deep learning technology in similar applications where a large-scale annotated dataset is unavailable because of the costly, tedious, and time-consuming nature of manual annotation. Our final model established a new state-of-the-art performance for wheat head segmentation.

### Domain adaptation

Domain adaptation methods were used to improve model performance in the presence of domain shift. There are three main domain shifts between the datasets used in this study.

1. The distribution shift between simulated dataset *S_t_* (*S_v_*) and the dataset *D_t_* (*D_v_*) that is generated by applying image transformations on real images: *D_t_* and *D_v_* were built by applying image transformations to real images. The choice of transformation has been made in a way that the manual annotation of the resulting images is possible. Unlike images in *S_t_* and *S_v_*, images in *D_t_* and *D_v_* show the whole structure of wheat plants and their positional characteristics. Therefore, images in *D_t_* and *D_v_* are semantically more similar to real images.

2. The distribution shift between the dataset *D_t_* (*D_v_*) and other real images in set Φ extracted from the wheat field video clip: The samples from D and Φ are frames that are extracted from the same wheat field video. While images in *D_t_* (*D_v_*) undergo extensive image transformations, images in Φ remain unchanged. Consequently, there is a distribution shift between images from these sets.

3. The distribution shift between the real image in Φ and images in Γ dataset: Images in Φ are from a single wheat field and a single plant growth stage. However, the images in Γ come from fields located in five different countries. This also introduces a domain shift between the image from Φ due to differences in the visual appearance of different wheat cultivars, background/soil appearance, and wheat developmental stage.

The model trained only using the synthesized datasets (model *S*) achieved a Dice score of 0.78 on the internal single-domain test set (Ψ) after TTA (Table 1). However, its performance dropped to 0.48 when evaluated on a diverse external dataset from 18 domains across five countries (Γ). This underscores the effect of domain shift between synthesized and real images. The substantial improvement in the performance measures after applying the first and second domain adaptation steps further highlights the utility of these domain adaptation steps in filling the gap between synthesized and real images.

Even after applying the second domain adaptation step, we still observed a drop in performance measures when we applied the resulting model (model *P*) to the external test set (Γ). This gap can be attributed to the domain shift between the images from a single domain used for model training and images from other domains, e.g., different growth stages or environmental conditions. This suggests that models trained on data from a single domain (e.g., a single field) might not generalize to external data (other fields). Further, internal evaluation using a test set that comes from the same distribution as the training set often does not provide a reliable estimate of generalization error. Using multidomain and diverse datasets for model evaluation should be considered the best practice for image-based plant phenotyping.

### Comparison to previous wheat head segmentation results

Our third test set (Λ) was developed in order to provide an indirect comparison to the supervised model developed by Rawat et al. [32], which is currently the state of the art in wheat head segmentation. Since we did not have access to the segmentation annotations for the UTokyo dataset, we randomly selected and annotated a subset of 100 images from this dataset for model evaluation. Our approach of utilizing only two annotated images (model *D*) achieved an average IoU of 0.71. This performance is similar to the average IoU reported by Rawat et al. [32], while using substantially fewer annotated images. It should be noted that Rawat et al. trained and evaluated their model on the UTokyo dataset; therefore, their evaluation is considered an internal evaluation. In contrast, our model evaluation using the Λ test set is regarded as an external evaluation since we did not use images from the UTokyo dataset for developing our models. External evaluation often provides a more reliable estimate of model performance and generalizability [47]. Further, the test set used by Rawat et al. and the test Λ used in this research are not the same. However, we expect our test set to be representative of the UTokyo dataset, providing a reliable performance measure. Our final model (model *G*), utilizing 38 GHWD annotated images (none from the UTokyo domain), after applying TTA achieved an IoU of 0.87 on the external test set (Λ), a random subset of images from the UTokyo domains of GWHD dataset. The proposed approach established—with about 16% IoU margin—a new state-of-the-art model for wheat head segmentation.

### Synthetic image generation

Generating synthetic crop images by compositing foreground objects on background scenes [37] has been explored in several prior works in image-based plant phenotyping, most notably for weed segmentation [48–50]. Many of these works have proposed advanced compositing approaches to generate higher-fidelity synthetic images that have a visual appearance closer to real images, such as employing gamma correction [49]. A novel aspect of our image synthesis pipeline is utilizing fake objects that have the shape of wheat head but the appearance of background regions (Fig. 6). In our preliminary analyses, we found that these negative samples were important for ensuring that the trained model did not overfit to contrast differences between the background scene and the foreground objects. Our results also show that injecting augmentations, both to foreground objects during the synthesis process and to the resulting composite images, enabled reasonable model performance with only two annotated video frames and with a simple direct overlay of foreground objects.

In this study, we used image frames from a single wheat field video. In this scenario, similar images are likely to be extracted because of the temporal coherence of the video clip and may reduce the variation of the training set. Therefore, we employed several different transformations and augmentations so that the final synthesized images will be different when presented to the model for training. For the internal evaluation, we selected images that are at least one second away from the frames used in the training set to avoid using consecutive image frames in training and test sets [51]. Further, to assess the generalizability of our approach, we performed model evaluation using the GWHD dataset, which is a diverse dataset due to differences in the visual appearance of different wheat cultivars, background/soil appearance, and wheat developmental stage.

### Limitations and future directions

In this study, we only used one short video clip of a wheat field to develop a deep semantic segmentation model. However, utilizing a larger number of video clips of wheat fields from various growth stages of the wheat could potentially further improve the performance of the model trained only using computationally annotated datasets.

In addition to the single wheat field video, we used 11 background scene videos in which there is no frame capturing wheat heads. In the data simulation step, we overlaid wheat heads onto image frames extracted from these background videos. By utilizing various background videos, we primarily intended to increase data variation reducing the chance of overfitting. We suggest a comprehensive study of the effect of various background videos on the model performance as future research.

We demonstrated that the proposed approach could lead to developing high-performing models for semantic segmentation of wheat heads. However, the application of the proposed method is not limited to the wheat head segmentation, and it could be used for other applications such as segmenting plants and plant organs in different crops where the aim is to segment highly self-similar patterns such as leaves, spikes, flowers, fruits, or diseased crops. This approach can also be extended to segment uncountable patterns and other phenotypic traits such as early vigor and canopy cover. However, it should be noted that while a single image could provide a good representation of a wheat field, it cannot represent general visual data such as the ImageNet dataset. Therefore, one should not expect to use one sample from the ImageNet dataset to provide a segmentation model for images in ImageNet.

The lack of publicly available annotated datasets is a challenge for evaluating wheat head segmentation models. The images and annotated segmentation masks utilized in this study will be made publicly available to enable reproduction and direct comparison by other researchers in future studies. We have also made the software for our image-synthesis pipeline open source. We hope that this will facilitate creating large-scale datasets by utilizing video clips from many fields and only annotating a single image from each clip. We suggest using a set of video clips representing various growth stages of the wheat for future research. One limitation of the annotated dataset used in this study is that it was generated by a single annotator. While this ensures that the annotation are self-consistent within the dataset, e.g., wheat head awns were excluded from the annotated wheat head regions, the annotations will be biased to the single annotator. As future work, we suggest developing additional benchmark datasets for future research to facilitate the evaluation of various models and to perform an evaluation of inter and intra-annotator variation in wheat head segmentation.

Although we used video data in this research, we did not leverage the temporal information between consecutive image frames, which has been previously explored for semantic segmentation in agriculture [52]. We propose extending our approach to utilize temporal information as future research.

Finally, in this study, we used a modified U-Net model architecture for all experiments. However, the proposed approach is independent of a specific model architecture. As future work, it would be interesting to evaluate how wheat head segmentation performance improves with more recent state-of-the-art segmentation architectures, such as [18], or if performance can be maintained when using lighter-weight segmentation architectures, such as [53], that could be deployed on edge devices directly in the field [54].

#### Conclusions

In this work, we introduced a semi-self-supervised approach for the wheat head semantic segmentation task by synthesizing a large-scale computationally annotated dataset and applying three domain adaptation steps to address challenges related to domain shift. Utilizing a few short video clips of a wheat field and background vegetation, the proposed method facilitates data collection for model building. Further, since the proposed model only uses a few manually annotated images, it avoids the manual annotation of a large dataset, making model faster and less costly to develop. While we showed the utility of the proposed method for wheat head segmentation, it could be applied to other applications that have similar dense repeating patterns of objects, such as segmenting plant organs in other crop species, or segmenting molecular components in microscopy images. This approach could also be extended to uncountable patterns such as estimating leaf area or canopy cover.

## Data Availability

Data used in this study are either publicly available or provided at https://www.cs.usask.ca/ftp/pub/whs/. The code for developing the synthesized dataset is available at https://github.com/KeyhanNajafian/ImageSimulatorPipeline.

## Supplementary Materials

Supplementary Materials Sections S1 to S3 Fig. S1
