# Supplementary material for: Semi-Self-Supervised Learning for Semantic Segmentation in Images with Dense Patterns
Source: Plant Phenomics. 2023 Feb 24;5:0025. doi: 10.34133/plantphenomics.0025 (PMC10013790; doi:10.34133/plantphenomics.0025)
Supplement: Supplementary Materials — Sections S1 to S3 Fig. S1 [file plantphenomics.0025.f1.pdf]

S1: Unsupervised, Self-supervised, and Semi-supervised Learning

Unsupervised, self-supervised, and semi-supervised learning methods have been developed to alleviate the need for large-scale annotated datasets [1]. In unsupervised learning, the goal is to learn a compact data representation using unannotated data. Semi-supervised learning [2] aims at utilizing both annotated and unannotated data for model development. Self-supervised learning refers to techniques that, instead of relying on manual annotation, utilize supervisory signals that are computationally generated from the data [3].

In self-supervised learning methods, a pretext task—which is often different from the primary task—is first defined. Image rotation [4], Image inpainting [5], and Jigsaw puzzle [6–8] are examples of pretext tasks. The pretext task is designed to generate a computationally-annotated dataset. Then a supervised approach is used to develop a model using the computationally-annotated dataset. The rationale behind self-supervised learning is that a model while learning the pretext task learns low-level features that can be shared across tasks. This model can then be fine-tuned to learn the primary task using a smaller amount of annotated data.

Contrastive learning [9, 10] is another category of self-supervised learning methods. Contrastive learning uses only the patterns and structure of unlabeled data to push positive input pairs closer together and negative input pairs farther apart, where a positive input pair are conceptually similar samples and a negative pair of samples are conceptually dissimilar. The majority of works in this domain differ in how positive and negative pairs are generated so as to minimize and maximize the distance between both groups, respectively. The conventional method for generating positive samples in the image domain involves augmenting an image in different ways.

S2: Image Augmentation

In this study, we benefit from a variety of image augmentations, including pixel-level and spatial-level transformations from the Albumentations package [11]. To synthesize S_t and S_v, real and fake wheat heads undergo a sequence of transformations including HorizontalFlip, VerticalFlip, Rotate, and ElasticTransform. After overlapping both real and fake wheat heads on the background images, we augment the resulting images using a long list of pixel-level transformations such as ColorJitter, ChannelShuffle, RGBShift, ChannelDropout, HueSaturationValue, Emboss, Solarize, InvertImg, ToGray, ToSepia, FancyPCA, Posterize, Sharpen, RandomGamma, Equalize, RandomBrightnessContrast, CLAHE, GaussianBlur, MotionBlur, RandomRain, RandomFog, RandomSnow, RandomSunFlare, GaussNoise, MultiplicativeNoise, ISONoise, and Normalize.

When generating D_t, D_v, in addition to the color transformations applied to the synthesized dataset, we included the following image augmentations: Flip, Rotation, ElasticTransform, GridDistortion, as well as a long list of RandomCrop functions with different squared crop sizes ranging from 400×400 to 1000×1000. These images then were resized to 1024×1024.

In the pseudo-labeling step, we tried all of the augmentation methods used when generating D_t and D_v (i.e., the first domain adaptation step) but excluded those that drastically changed the color, like ColorJitter, Channel Shuffle, and RGB Shift. In the last training step, we also utilized the same list applied in the first domain adaptation step to augment the chosen training samples from the GWHD dataset. During model evaluation, only the Resize, and Normalize transformations were applied to images.

S3: Model Architecture and Hardware Specification

For all experiments, we have used a Tesla V100S-PCIE-32GB GPU device to develop all our models. The machine uses a Intel(R) Xeon(R) Gold 5220 CPU @ 2.20GHz CPU with 72 visual cores.

```
===================================================================================================================
Layer (type:depth-idx)                                       Output Shape              Param #
===================================================================================================================
Unet                                                         [4, 1, 1024, 1024]        --
├─EfficientNetEncoder: 1-1                                   [4, 3, 1024, 1024]        806,400
│    └─Conv2dStaticSamePadding: 2-1                          [4, 48, 512, 512]         1,296
│    │    └─ZeroPad2d: 3-1                                   [4, 3, 1025, 1025]        --
│    └─BatchNorm2d: 2-2                                      [4, 48, 512, 512]         96
│    └─MemoryEfficientSwish: 2-3                             [4, 48, 512, 512]         --
│    └─ModuleList: 2-4                                       --                        --
│    │    └─MBConvBlock: 3-2                                 [4, 24, 512, 512]         2,940
│    │    └─MBConvBlock: 3-3                                 [4, 24, 512, 512]         1,206
│    │    └─MBConvBlock: 3-4                                 [4, 32, 256, 256]         11,878
│    │    └─MBConvBlock: 3-5                                 [4, 32, 256, 256]         18,120
│    │    └─MBConvBlock: 3-6                                 [4, 32, 256, 256]         18,120
│    │    └─MBConvBlock: 3-7                                 [4, 32, 256, 256]         18,120
│    │    └─MBConvBlock: 3-8                                 [4, 56, 128, 128]         25,848
│    │    └─MBConvBlock: 3-9                                 [4, 56, 128, 128]         57,246
│    │    └─MBConvBlock: 3-10                                [4, 56, 128, 128]         57,246
│    │    └─MBConvBlock: 3-11                                [4, 56, 128, 128]         57,246
│    │    └─MBConvBlock: 3-12                                [4, 112, 64, 64]          70,798
│    │    └─MBConvBlock: 3-13                                [4, 112, 64, 64]          197,820
│    │    └─MBConvBlock: 3-14                                [4, 112, 64, 64]          197,820
│    │    └─MBConvBlock: 3-15                                [4, 112, 64, 64]          197,820
│    │    └─MBConvBlock: 3-16                                [4, 112, 64, 64]          197,820
│    │    └─MBConvBlock: 3-17                                [4, 112, 64, 64]          197,820
│    │    └─MBConvBlock: 3-18                                [4, 160, 64, 64]          240,924
│    │    └─MBConvBlock: 3-19                                [4, 160, 64, 64]          413,160
│    │    └─MBConvBlock: 3-20                                [4, 160, 64, 64]          413,160
│    │    └─MBConvBlock: 3-21                                [4, 160, 64, 64]          413,160
│    │    └─MBConvBlock: 3-22                                [4, 160, 64, 64]          413,160
│    │    └─MBConvBlock: 3-23                                [4, 160, 64, 64]          413,160
│    │    └─MBConvBlock: 3-24                                [4, 272, 32, 32]          520,904
│    │    └─MBConvBlock: 3-25                                [4, 272, 32, 32]          1,159,332
│    │    └─MBConvBlock: 3-26                                [4, 272, 32, 32]          1,159,332
│    │    └─MBConvBlock: 3-27                                [4, 272, 32, 32]          1,159,332
│    │    └─MBConvBlock: 3-28                                [4, 272, 32, 32]          1,159,332
│    │    └─MBConvBlock: 3-29                                [4, 272, 32, 32]          1,159,332
│    │    └─MBConvBlock: 3-30                                [4, 272, 32, 32]          1,159,332
│    │    └─MBConvBlock: 3-31                                [4, 272, 32, 32]          1,159,332
│    │    └─MBConvBlock: 3-32                                [4, 448, 32, 32]          1,420,804
│    │    └─MBConvBlock: 3-33                                [4, 448, 32, 32]          3,049,200
├─UnetDecoder: 1-2                                           [4, 32, 1024, 1024]       --
│    └─Identity: 2-5                                         [4, 448, 32, 32]          --
│    └─ModuleList: 2-6                                       --                        --
│    │    └─DecoderBlock: 3-34                               [4, 512, 64, 64]          5,163,008
│    │    └─DecoderBlock: 3-35                               [4, 256, 128, 128]        1,899,520
│    │    └─DecoderBlock: 3-36                               [4, 128, 256, 256]        479,744
│    │    └─DecoderBlock: 3-37                               [4, 64, 512, 512]         138,496
│    │    └─DecoderBlock: 3-38                               [4, 32, 1024, 1024]       27,776
├─SegmentationHead: 1-3                                      [4, 1, 1024, 1024]        --
│    └─Conv2d: 2-7                                           [4, 1, 1024, 1024]        289
│    └─Identity: 2-8                                         [4, 1, 1024, 1024]        --
│    └─Activation: 2-9                                       [4, 1, 1024, 1024]        --
│    │    └─Identity: 3-39                                   [4, 1, 1024, 1024]        --
===================================================================================================================
Total params: 25,257,449
Trainable params: 25,257,449
Non-trainable params: 0
Total mult-adds (G): 596.74
===================================================================================================================
Input size (MB): 50.33
Forward/backward pass size (MB): 19675.48
Params size (MB): 31.32
Estimated Total Size (MB): 19757.13
===================================================================================================================
```

Figure S1: The architecture of the customized U-Net model used in this study.

References

[1] L. Schmarje, M. Santarossa, S.-M. Schröder, and R. Koch, "A survey on semi-, self- and unsupervised learning for image classification," *IEEE Access*, vol. 9, pp. 82 146–82 168, 2021.

[2] X. J. Zhu, "Semi-supervised learning literature survey," 2005.

[3] Y.-H. H. Tsai, Y. Wu, R. Salakhutdinov, and L.-P. Morency, "Self-supervised learning from a multi-view perspective," *arXiv preprint arXiv:2006.05576*, 2020.

[4] N. Komodakis and S. Gidaris, "Unsupervised representation learning by predicting image rotations," in *International Conference on Learning Representations (ICLR)*, 2018.

[5] D. Pathak, P. Krahenbuhl, J. Donahue, T. Darrell, and A. A. Efros, "Context encoders: Feature learning by inpainting," in *Proceedings of the IEEE Conference on Computer Vision and Pattern Recognition*, 2016, pp. 2536–2544.

[6] M. Noroozi and P. Favaro, "Unsupervised learning of visual representations by solving jigsaw puzzles," in *European Conference on Computer Vision*, Springer, 2016, pp. 69–84.

[7] C. Wei *et al.*, "Iterative reorganization with weak spatial constraints: Solving arbitrary jigsaw puzzles for unsupervised representation learning," in *Proceedings of the IEEE/CVF Conference on Computer Vision and Pattern Recognition*, 2019, pp. 1910–1919.

[8] D. Kim, D. Cho, D. Yoo, and I. S. Kweon, "Learning image representations by completing damaged jigsaw puzzles," in *2018 IEEE Winter Conference on Applications of Computer Vision (WACV)*, IEEE, 2018, pp. 793–802.

[9] T. Chen, S. Kornblith, M. Norouzi, and G. Hinton, "A simple framework for contrastive learning of visual representations," in *International Conference on Machine Learning*, PMLR, 2020, pp. 1597–1607.

[10] K. He, H. Fan, Y. Wu, S. Xie, and R. Girshick, "Momentum contrast for unsupervised visual representation learning," in *Proceedings of the IEEE/CVF Conference on Computer Vision and Pattern Recognition*, 2020, pp. 9729–9738.

[11] A. Buslaev, V. I. Iglovikov, E. Khvedchenya, A. Parinov, M. Druzhinin, and A. A. Kalinin, "Albumentations: Fast and flexible image augmentations," *Information*, vol. 11, no. 2, 2020, ISSN: 2078-2489. DOI: 10.3390/info11020125. [Online]. Available: https://www.mdpi.com/2078-2489/11/2/125.
